# Psychodrama Group Therapy for Social Issues: A Systematic Review of Controlled Clinical Trials

**DOI:** 10.3390/ijerph18094442

**Published:** 2021-04-22

**Authors:** M. Angeles López-González, Pedro Morales-Landazábal, Gabriela Topa

**Affiliations:** 1 Department of Psychology/Faculty of Health Sciences, Universidad Rey Juan Carlos, Avenida de Atenas s/n, Alcorcón, 28040 Madrid, Spain; angeles.lopezg@urjc.es; 2 Independent Scholar, 28016 Madrid, Spain; pemolasii@yahoo.es; 3 Department of Social and Organizational Psychology, National University of Distance Education, 28040 Madrid, Spain

**Keywords:** psychodrama group therapy, systematic review, randomized controlled trials

## Abstract

The aim of this study was to carry out a systematic review of controlled clinical trials in order to identify both specific populations and social issues which may benefit from the effective use of psychodrama psychotherapy. A search was conducted in the WoS, SCOPUS, PsychINFO, Medline, Academic Search Ultimate, ProQuest, and PubPsych databases, complemented by a manual search on relevant websites and in the reference lists of the selected studies. Randomized controlled trials (RCTs) and quasi-RCTs of group-based psychodrama psychotherapy were included. The Effective Public Health Practice Project (EPHPP) tool was adopted to assess the methodological quality of the included studies. The search identified 14 RCTs and one quasi-RCT evaluating the effects of group-based psychodrama psychotherapy. The total number of participants in the studies was 642 people. Seven studies were conducted in Turkey, two in the USA, two in Finland, one in Canada, one in Brazil, one in Italy, and one in Iran. The heterogeneity of the issues analyzed indicates that psychodrama improves the symptoms associated with a wide range of problems. Despite psychodrama’s long history, most clinical trials in this field have been published this century, which suggests not only that this psychotherapeutic practice remains relevant today but also that it continues to attract substantial interest among the scientific community. Nevertheless, further research efforts are required to understand its potential benefits for psychosocial well-being.

## 1. Introduction

The mental health and psychological care programs run at hospitals all over the world have been improved and updated over recent years. It has been observed that classic psychotherapeutic interventions designed to foster health often trigger significant psychological changes that, although sustained during the first few months, later disappear gradually over time [1]. Consequently, and in accordance with the principles of the biopsychosocial approach, experts are currently aiming to provide ongoing, comprehensive care to patients in keeping with more efficient, holistic models [2]. This type of care is also vital to responding to emerging social issues, particularly among vulnerable populations. In these cases, a number of treatments based on group therapy have been established, one of which is psychodrama [3,4,5,6,7,8].

Psychodrama, first created by Jacob Levy Moreno in 1921, is a type of psychotherapy initially conceived as “deep group psychotherapy”, which was inspired by improvisation theater. The key aspect of psychodrama is the dramatization by patients of a series of events as if they were happening in the present. During psychodrama interventions, emphasis is placed not only on what patients say but also on what they do (the action) during the dramatization. The aim is for patients to gain greater insight into their situation in order to enable them to cope better with their thoughts and feelings and increase their personal resources and strengths [9]. This psychotherapeutic technique offers a broad range of possibilities when applied to mental healthcare programs, and reports have been published of effective interventions with clinical patients suffering from different psychological disorders, e.g., [10,11] and people with subclinical symptoms, e.g., [12,13] and as part of personal development plans aimed at, for example, improving social skills, boosting self-esteem or changing attitudes, e.g., [14,15]. Another set of interesting studies are those carried out at a community level focusing on interdisciplinary prevention programs implemented in schools, e.g., [16,17].

Psychodrama employs five principal elements [18,19]: (a) protagonist, someone from the group who acts out, in different scenes, roles linked to possible difficulties and/or personal potentialities; unlike in real theater, the actor is also the author of their own work; (b) auxiliary egos, qualified helpers who play the role of co-therapists; they direct the protagonist while at the same time acting as external observers of the scene being played out; their purpose is to play complementary roles; (c) director, who acts as both therapist and analyst; the director is responsible for guiding the session and for monitoring the progress of the psychotherapeutic process; they are therefore responsible for choosing the most suitable therapeutic strategy and using different psychodrama techniques as required; (d) audience, made up of the other members of the group; the audience may help by serving as a “sounding box”, amplifying or reinforcing the protagonist’s sensations and acknowledging and understanding their experience, thereby helping everyone recognize their own conflicts; and (e) stage, a specific space in which the scene takes place and is acted out; the stage enables the protagonist to represent their inner world and to play out all their dreams and fantasies, thereby becoming the “space of the possible”.

According to Moreno’s classic psychodrama, each session comprises three phases [9,19]: warming up, action, and sharing. (a) Warming up is the preparation stage in which initial contact is established between the director and the members of the group; during this stage, the aim is to foster group interaction and engage in exercises designed to gradually increase spontaneity, reduce inhibitions to sharing one’s own experiences and increase participants’ engagement in the session. (b) The action phase (enactment) is the moment in which the dramatic action (the core aspect of psychodrama) takes place; the protagonist goes onto the stage and acts out their symbolic or real scene; then, a series of other scenes are acted out which simulate real-life situations and reflect past events, present challenges or future possibilities; in other words, everything that is worrying the protagonist is represented on stage; important events in their past are explored, giving them the opportunity not only to recount and experience them but also to act on them and reintegrate them in a new way, thus searching for a resolution to the conflict posed; the aim is to break away from preconceptions, to deconstruct and then reconstruct the patient’s internal elements in a process known as catharsis or rational and emotional mobilization. Finally, (c) sharing, is the phase in which the group shares the experiences and emotions triggered by the session, with no rational interpretations or questions being allowed; as a result, the patient (and sometimes various members of the group) may find a way to emotionally or cognitively integrate their world.

The first academic studies on psychodrama were carried out by its original developer, Moreno [20,21,22]. Although these initial papers were followed by many others, traditionally, the practitioners of psychodrama have generally tended to publish either descriptive studies based on specific cases or theoretical discussions [23,24]. Over recent decades, several reviews have attempted to sum up the findings of research in this field [24,25,26,27,28,29,30,31]. Thus, the review by Kellermann [32] focused on the results published between 1952 and 1985, analyzing 23 studies including both controlled trials in which psychodrama was not necessarily the only treatment given and trials aimed at validating only one particular psychodrama technique. Kellermann concluded that psychodrama is an effective therapeutic alternative for fostering behavioral changes in adjustment, antisocial and related disorders.

Sometime later, the meta-analysis by Kipper and Ritchie [25] reviewed trials published between 1965 and 1999. The efficacy of certain psychodrama techniques was confirmed both at an individual level and when all were assessed as a whole. The analyses revealed an effect size of 0.95 (slightly higher than that established in the scientific literature for group therapy), and moreover, the techniques of role reversal and doubling emerged as the most effective. More than focusing on the whole session (which would be the classic approach), the authors aimed to highlight the importance of psychodrama techniques, pointing out that each of them plays a key role in the process as a whole.

The contributions made by Weiser are also important. In 2006, this author [27] analyzed the results of a meta-analysis carried out by Grawe some years earlier (1994), confirming that psychodrama is effective for treating neurosis and schizophrenia. Nevertheless, the review concluded by stating that the main therapeutic benefits of psychodrama were on patients’ interpersonal relationships and that the effect on their symptoms was weaker. One year later, the same author published another review [29] which encompassed over 50 studies on (a) specific techniques that can be applied during psychodrama interventions; (b) results from a variety of clinical trials involving different drama therapies; and (c) the efficacy of the psychotherapeutic process.

Another work that aimed to highlight the value of psychodrama techniques was the systematic review conducted by Cruz et al. [30]. As well as identifying the different techniques used in Moreno’s psychodrama, the aim of this review was to determine which enjoyed the highest level of consensus among the scientific community. A total of 11 principal techniques were identified, namely soliloquy, double, mirror, role reversal, resistance interpolation, sculpture, social atom, intermediate objects, games, sociometry, and role training.

Finally, in 2019, Orkibi and Feniger-Schaal [24] carried out another systematic review covering research using diverse methodologies (qualitative, case studies, controlled trials, single-group designs, etc.) published during the first decade of the 21st century. The review did not focus exclusively on psychodrama, but rather included all methods which use drama as a therapeutic tool, including drama therapy. The authors highlighted the fact that while research into psychodrama interventions has increased over the years, the methodological quality of some of the studies carried out should be improved. Thus, due to greater pressure to ensure evidence-based interventions and decrease susceptibility to subjective interpretation, over recent decades, an increasingly number of studies have begun to document psychotherapeutic interventions based on psychodrama [24,33].

There are several problems associated with controlled research, such as the definition of areas of therapeutic efficacy, the specific details of the treatment provided, and the validation of the approach [34], which is why it is so important to operationalize the expected changes by measuring specific results and implementing solid research designs [34]. As a result of these difficulties, the reviews published in this field all suffer from some kind of conceptual, temporal, or methodological limitation [24]. For example, the aforementioned meta-analysis by Kipper and Ritchie [25] only includes works published up until the turn of the century, and its main aim was to assess the efficacy of different psychodrama techniques and other reviews, such as the one by Castelo et al. [28], adopt a regional approach. Finally, there are also other reviews in which psychodrama forms part of a multi-component intervention alongside other treatments, making it impossible to assess its efficacy in an independent manner [29,35,36,37,38].

The results in relation to efficacy areas are unclear, particularly given that the definition of psychodrama differs from one study to another. In some cases, for example, controlled research includes publications focusing on dramatherapy, using the two terms synonymously. The differences between drama therapy and psychodrama are clear since although they share some fundamental aspects, psychodrama works with real representations acted out on the stage, whereas drama therapy is based on metaphor through art and uses personal scenes that are both fictitious and symbolic. Moreover, in dramatherapy, patients usually participate in the narrative in a more indirect manner and with greater distance [39]. On other occasions, it is assumed that the results of research into one single psychodrama technique, such as role-playing [34] or role-taking [40], reflect the entire psychotherapeutic process involved in psychodrama when in reality, they are only instruments to be used in specific situations by the psychodrama therapist.

To evaluate the changes achieved by the psychodrama therapeutic model, we must examine studies that use the classic group format (psychodrama group therapy), with no complementary individual interventions, in which it is possible to clearly identify the warming up, action, and sharing phases established by Moreno himself (classic psychodrama, see [32]). Furthermore, to analyze whether psychodrama is a true agent for change, the outcome measures should be capable of assessing specific changes in patients, and studies should be methodologically rigorous, comparing at least one experimental group (treated only with psychodrama) with a control group. Thus, the general aim of this present systematic review is to identify, within the framework of previous publications, evidence of psychotherapeutic interventions based on “classic psychodrama” that adhere to a rigorous research design (controlled clinical trials). The specific aims are: (a) to determine the scientific production and quality of the selected studies; (b) to identify the problems treated; and (c) to analyze the methodological characteristics of the research carried out.

## 2. Materials and Methods

Following the method used in previous studies, e.g., [41,42,43], this review implemented a series of control mechanisms designed to reduce any bias that may exist a priori, as suggested by the PRISMA—preferred reporting items for systematic reviews and meta-analyses—method, e.g., [44,45]. The study was registered in the International Prospective Register of Systematic Reviews (PROSPERO) in 2021, and the detailed prespecified protocol is available on request. We, therefore, developed a protocol that enabled us to apply criteria in a uniform manner to all publications, including search terms, established time limit, search timing, and inclusion of the database selection.

### 2.1. Procedure

The documents included were journal articles and doctoral theses, following the guidelines established for evidence-based reviews by Martín, Garcés, and Seoane [46], who propose that gray literature be included in order to avoid publication bias.

During the search, we followed the recommendations made by Sánchez-Meca, Marín-Martínez, and López-López [47], who suggest a combination of formal and informal search strategies: (a) formal searches were conducted in the computerized thematic databases PsycINFO, PubPsych, and Medline, as well as in the multidisciplinary databases Scopus, Academic Search Ultimate, Core Collection of Web of Science, and Proquest Research Library; and (b) informal searches were conducted for relevant works extracted from the analysis of the bibliographic references of journal articles.

The selected works adhered to the following search terms: (TITLE-ABS-KEY (psychodrama) AND TITLE-ABS-KEY (control* OR random* OR trial OR “comparison group” OR groups OR effective* OR “therapeutic efficacy”) AND NOT TITLE-ABS-KEY (“drama$therapy” OR “case study” OR “single case” OR “case report” OR “review” OR “scene-based psychodramatic family therapy” OR “theatre-based interventions” OR “drama$programme” OR “Improvisation Theater” OR “Improvisation theatre” OR “theatre activity” OR “drama group therapy”).

Additional works were identified in the references of all the publications returned by the search, including relevant meta-analyses and systematic reviews. Publications featured in the Journal of Psychodrama Sociometry and Group Psychotherapy were reviewed manually since this journal is published by the American Society of Group Psychotherapy and Psychodrama. Moreover, the bibliographic entries featured on the website http://pdbib.org/ (accessed on 20 April 2020), compiled by James M. Sacks and Michael Wieser, were also reviewed, and contact was made with key authors to locate some original works we were unable to find and to ask them for information on any unpublished studies of interest.

The respective documentary searches were carried out by MALG in April 2020. The bibliographic references of the different works were analyzed and the studies included in the prior reviews at the same time as we continued with the more formal search procedure. No language restrictions were established, and all publications dated up until December 2019 were included.

Two authors (MALG and PML) reviewed the results returned by the search and extracted those references that complied with the pre-established inclusion criteria. Inter-rater reliability between the two reviewers was measured using the Kappa statistic (k = 0.81; 95% CI: 0.75–0.87). Any doubts were resolved by consulting a third member of the team (GT). All potentially relevant studies were retrieved so that their full texts could be read.

### 2.2. Inclusion and Exclusion Criteria

Only those controlled trials in which an experimental group exposed to a treatment based exclusively on group psychodrama psychotherapy was compared to another group that acted as either an active (other treatment) or passive (waiting list and/or placebo) control group were considered. We followed Munn, Stern, Aromataris, Lockwood, and Jordan [48], who recommend that, in systematic reviews on efficacy, the classic PICOS [48] (participants, interventions, comparisons, outcomes, and study designs) format be used. Inclusion criteria were as follows: (a) participants, no limits were established as regards age (i.e., minors, adults, people over 65 years of age), sex or number of participants, and nor were any restrictions imposed regarding suffering (or not) from a pathology of any kind or having been (or not) admitted to some kind of mental health center; (b) intervention, only empirical works researching the efficacy of a treatment based on the classic group psychodrama psychotherapy format were included; (c) comparisons, only experimental and quasi-experimental clinical trials comparing different groups were included; this included both two-group (experimental and control) and multi-group designs with a control group (active or passive); studies without a control group and case studies were excluded; (d) outcomes, only those studies which included pre and post-test measures, carried out using validated assessment instruments with evidence of reliability were included (the dropout rate during psychotherapy should be noted and stated, although this was not one of the selection criteria established for inclusion in the review); and (e) study design: randomized controlled trials (RCTs) and quasi-randomized controlled trials were deemed eligible.

Exclusion criteria included methodological aspects and factors relating to the intervention itself. Thus, reviews, theoretical works, case studies, single-group trials, and those in which group psychodrama formed part of a multi-treatment package [49,50,51,52,53] were not deemed eligible for inclusion; and qualitative works [54] which did not report statistical data or those whose data were obtained using non-validated instruments were also excluded. Furthermore, previously structured interventions, e.g., [55,56], were excluded. By this, we mean any procedure in which the intervention was “rendered in a structured format which was repeated for each subject rather than the highly spontaneous manner which characterizes Moreno’s original method” (Kipper and Giladi [55], p. 501), along with those which failed to specify the three classic phases (i.e., warming up, action, and sharing), e.g., [57,58], and those that complemented group psychodrama psychotherapy with individual psychotherapy, e.g., [57].

### 2.3. Coding the Publications

During the first phase, each reference was analyzed individually to eliminate false positives and irrelevant records. A customized database was generated, creating a spreadsheet accessible in the cloud.

A quality assessment tool for quantitative studies (Effective Public Health Practice Project, EPHPP [59,60]) was used to further explore the works’ suitability. Quality assessment of the studies was conducted by two members of the research group. Discrepancies were resolved by consensus, resulting in the following fields being included in the database compiled by MALG and PML: (a) authorship of the document; (b) year of publication; (c) title of the publication; (d) selection bias (i.e., information related to the representativeness of the sample); (e) study design (i.e., information about the randomization of the subjects to the experimental conditions); (f) confounders (i.e., controlling for strange variables); (g) blinding (single or double bind); (h) data collection method; (i) withdrawals and drop-outs (percentage of participants completing the study during the final data collection period in all groups: enrollment, allocation, follow-up, and analysis); (j) intervention integrity (e.g., the percentage of participants who completed the intervention and its consistency, measured in terms of whether or not the intervention was provided to all participants in the same way and the level of therapist competence); and (k) analyses (e.g., unit of allocation, unit of analysis, statistical methods appropriate for the study design, analysis performed by intervention allocation status (i.e., intention to treat) rather than the actual intervention received).

## 3. Results

### 3.1. Scientific Production and Quality of the Publications

**Figure 1:**
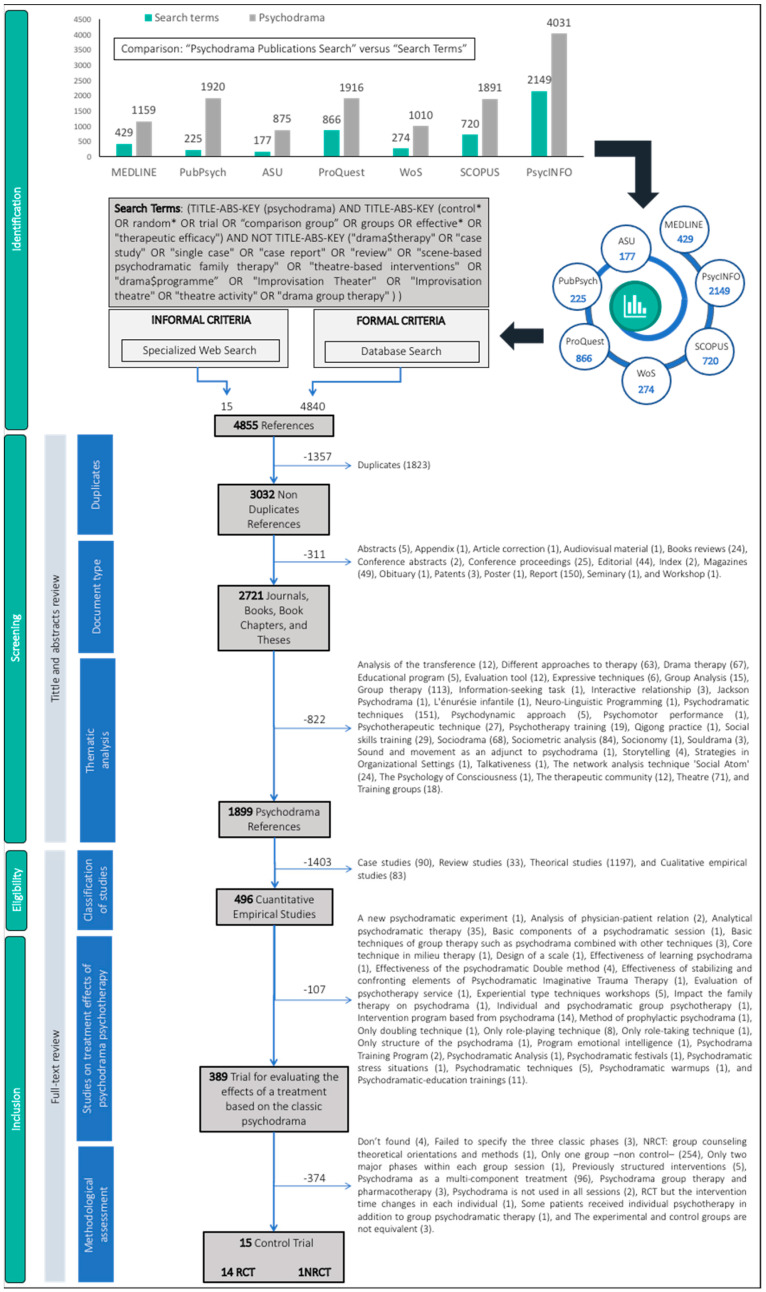
Flow of information through the different phases of the systematic review. Note: TI (title), AB (abstract), KW (keywords), RCT (randomized controlled trial), q-RCT (quasi-randomized controlled trial).

The graph at the top of Figure 1 shows the results of the document search, comparing the records pertaining to the selected search terms with those obtained by including only the term “psychodrama”. As shown in the graph, 26.45% of all items on psychodrama (4840 items) published up until December 2019 included the selected terms. At the bottom of Figure 1 is a flow diagram charting the process followed for retrieving the relevant works. The process begins by specifying the number of references extracted from each database searched. The diagram also specifies the number of documents obtained throughout the two phases of the process. Firstly, the results for the initial phase (reading of the titles and abstracts) are given, indicating how many duplicates and non-relevant references (due to either type of document or topic) were removed. Secondly, the diagram specifies the number of references recovered during the final phase (i.e., the reading of the full texts). The decision to include or exclude references depended on the type of study they were (empirical works) and their design (controlled trials). Following the manual analysis, a total of 15 controlled trials were selected, representing 0.49% of all non-duplicated references returned by the search. As regards experimental conditions, 12 studies assessed the effects of psychodrama psychotherapy in comparison with a passive control group (i.e., waiting list), and the remaining three did so in comparison with an active control group (i.e., other treatment). As for study design, 14 were randomized controlled trials, and one was a quasi-randomized controlled trial.

Table 1 presents the bibliometric data pertaining to the retrieved references, indicating the year of publication for each document, the authors’ full names, affiliation and country, type of document, and, in the case of articles, the name of the journal and its quality index. As shown in Table 1, 44 different signatures were identified corresponding to 37 different authors, with the most prolific being Zeynep Karataş (4), Turkan Dogan (2), Zafer Gökçakan (2), Kari Kähönen (2), and Katariina Salmela-Aro (2). Moreover, 66.66% of the references were co-authored, with the collaboration index being 2.73. As for international scope, the publications were written by authors from 11 independent research teams with affiliations in Turkey (7), the USA (2), Finland (2), Canada (1), Brazil (1), Italy (1), and Israel (1). Collaborative works were ascribed to a single country in all cases, meaning that no international collaboration was detected.

Finally, as regards the quality of the publications, Table 2 shows the database compiled on the basis of the EPHPP criteria [59]. In terms of the final score, nine of the studies obtained a general global rating of “strong”, and seven obtained the category “moderate” [61,62,63,64].

### 3.2. Problems Treated

Table 3 presents the results of psychodrama interventions applied to different groups of problems, with varied samples. The themes are categorized in accordance with the type of participant: those with clinical and subclinical symptoms and community samples. Firstly, in the four controlled trials carried out with clinical patients, the problems dealt with were psychic suffering and behavioral disorders (mood disorders, suicide attempts, psychotic episodes, eating disorders, and social isolation) [63], oppositional defiant disorder [65], opioid dependence [61], and Parkinson’s disease [66]. Psychodrama proved effective for improving the symptoms of the oppositional defiant disorder; specifically, participants displayed a greater impulsive response latency and a lower frequency of oppositional behaviors [65]. In the other controlled trials carried out with clinical patients, the dependent variable was not related to the symptoms of their disorder but rather to their biopsychosocial adjustment. Thus, improvements were observed in psychological distress [63], quality of life [66], and perceived health [61].

Seven of the randomized controlled trials were carried out with patients with subclinical symptoms. In these studies, the intervention aimed to treat different social issues linked to lack of control over aggressive impulses [62,67,68,69], traumatic experiences [70], and chronic work-related stress or burnout [71,72]. For example, Smokowski and Bacallao [69] carried out an intervention aimed at preventing youth violence in a family context with Latin American migrants. Parents reported an improvement in their children’s defiant, oppositional behavior, as well as a decrease in parent-child conflict. Following the intervention, participants were better able to express their feelings and had a better knowledge of themselves. They also reported more optimistic attitudes [72] and greater use of creative solutions [71]. The effects of psychodrama therapy on burnout were explored by the team led by Kähönen in two articles using two modulating variables: sense of coherence [73] and eudaimonic psychological well-being [71]. In both studies, Kähönen et al. recruited public sector workers (e.g., police officers and public prosecutors) with severe symptoms of burnout. Eight treatment groups were created (four psychodrama groups and four analytic groups) along with a control group; the intervention lasted nearly nine months. In the article on sense of coherence, the psychodrama groups were found to improve more quickly during the intervention, although recovery in the analytic groups lasted longer. The changes observed in the study on psychological well-being also affected the control group. However, as in the other study by the same team, these changes were found to occur more quickly in the psychodrama groups but were more stable during follow-up in the analytic groups.

Finally, four clinical trials were carried out with community samples. The aim was to help participants develop professional competencies [72,74] or personal strengths that would have a positive impact on their mental health [64,75]. Thus, Özbas and Tel [72] implemented a program of psychodrama treatment designed to improve the quality of professional care provided by oncology nurses. The aim of the program was to increase perceived levels of psychological and work-related empowerment and decrease burnout levels among nursing staff. The results reported by Dogan [74] were positive, with psychodrama being found to instill professional competencies such as empathy and self-awareness in students enrolled in a psychological counseling and guidance course. In a previous study, this same author [64] analyzed the consequences of an intervention on romantic attachment among adults. The aim was to explore the benefits of psychodrama for fostering secure and healthy attachment behaviors. The results revealed significant improvements in one of the dimensions evaluated: anxiety, measured after the treatment, although no differences were observed between the experimental and control groups. Finally, Karatas [75] explored the importance of studying hopelessness as a variable that may influence the development of certain psychological problems (e.g., depression, ideas of suicide, etc.) if sustained over time. The author confirmed the significant positive effect of group psychodrama on subjective well-being and hopelessness.

**Table 3:** Samples, variables, measures, and outcomes of psychodrama interventions applied to different groups.

Study	Sample	Variables	Measures	Outcomes	
Carbonell y Parteleno-Barehmi, 1999 [70]	Subclinical	Depression, verbal aggression, delinquent behavior, thought problems, somatic complaints, social (withdrawn) problems, attention-seeking behaviors, and phobic-anxious behavior	YSR	PD/CG post: Withdrawn: F = 10.47 *; anxious/depressed: F = 5.97 *; somatic: F = 1.04; social problems: F = 4.02; thought problems: F = 2.88; Attention problems: F = 0.14; delinquent behavior: F = 1.96; aggressive: F = 3.72.	Pre/Post
Singal, 2003 [65]	Clinical	Impulsivity, empathy, self-esteem, oppositional behaviors	MFFT BEES SEI CRS-R JI	PD/CG post: Latency response: F = 7.69 *; error: F = 0.46; empathy: F = 1.78; self-esteem: F = 0.56; CRS-R, parents’ reports: oppositional subscale: F = 6.07 *, cognitive problems/inattention subscale: F = 0.04, hyperactivity subscale: F = 0.57, and ADHD index subscale: F = 1.47. CRS-R, teachers’ reports: oppositional subscale: F = 11.83 *, cognitive problems/inattention subscale: F = 0.21, hyperactivity subscale: F = 1.13, and ADHD index subscale: F = 0.54.	Pre/Post
Karataş y Gökçakan, 2009a [67]	Subclinical	Physical aggression, verbal aggression, anger, hostility, indirect aggression, total aggression	Aggression Scale	CBT/CG. Total aggression: F = 117.092 *; physical aggression: F = 37.74 *; anger: F = 50.04 *; hostility: F = 27.23 *; indirect aggression: F = 24.04 *. PD/CG post: Total aggression: F = 65.10 *; anger: F = 20.17 *; hostility: F = 18.59 *; indirect aggression: F = 40.99 *. CBT/PD post: Total aggression: F =15.22 *; physical aggression: F = 28.02 *; anger: F = 10.67 * Non-significant differences; post-test/follow-up in both experimental groups.	Pre/Post/3-month follow-up
Karataş y Gökçakan, 2009b [68]	Subclinical	Physical aggression, verbal aggression, anger, hostility, indirect aggression, total aggression	Aggression Scale	Total aggression: F = 65.11 ***; physical aggression: F = 3.38; verbal aggression: F = 1.85; anger: F = 20.17 ***; hostility: F = 18.59 ***; indirect aggression: F = 40.99 *** Non-significant differences; post-test/follow-up in P group	Pre/Post/3-month follow-up
Smokowski y Bacallao, 2009 [69]	Subclinical	Parent-adolescent conflict, oppositional defiant problems, anxious-depressed problems	CBCL CBQ-20	Oppositional defiant behavior: F = 5.50 ***; Anxious-depressed behavior: F = 3.80 **; Parent-adolescent conflict: F = 4.10 ***; Total problems: F = 3.30 *	Pre/12-month follow-up
Dogan, 2010 [64]	Community	Anxiety, avoidance	ECR-R	PD pre/post: Anxiety: *z* = −2.36 *; Avoidance: *z* = −1.51. CG pre/post: Anxiety: *z* = −1.60; Avoidance: *z* = −0.80.	Pre/post
Gatta et al., 2010 [63]	Clinical	Psychic suffering and behavioral disorders	SCL-90-R	PD pre/post: SOM: *z* =−2.02 *; O-C: *z* = −0.53; IntSens: *z* = −0.41; DEP: *z* = −1.16; ANX: *z* = −1.63 *; HOS: *z* = −1.08; PHOB: *z* = −0.63; PAR: *z* = −1.35; PSY: *z* = −2.03 *; GSI: *z* = −1.99 *. GC pre/post: SOM: *z* =−1.35; O-C: *z* = −0.27; IntSens: *z* = −0.54; DEP: *z* = −0.67; ANX: *z* = −0,67; HOS: *z* = −0.55; PHOB: *z* = −0.36; PAR: *z* = −0.73; PSY: *z* = 0.00; GSI: *z* = −0.52.	Pre/post
Sproesser et al., 2010 [66]	Clinical	Depression, anxiety and Parkinsonism symptoms, systemic symptoms, emotional functioning, and social functioning	BDI STAI PDQL	Depression. PD, pre (M = 23, StD = 12), post (M = 9, StD = 9); CG, (M = 11, StD = 6), post (M = 12, StD = 6) ** Anxiety. PD, pre (M = 49, StD = 11), post (M = 34, StD = 11); CG, pre (M = 43, StD = 12), post (M = 46, StD = 14) ** Quality of life. PD, pre (M = 95, StD = 16), post (M = 77, StD = 22); CG, pre (M = 94, StD = 31), post (M = 97, StD = 27) *.	Pre/post
Karataş, 2011 [62]	Subclinical	Aggression, problem solving	CRBDS	PD/CG post: Aggression: U = 17.00 *; Problem solving: U = 2.50 *. PD/IG post: Aggression: U = 28.00 *; Problem solving: U = 0.00 *.	Pre/post/ 2.5-month follow-up
Kähönen et al. 2012 [73]	Subclinical	Sense of coherence	OLQ-13	PD/CG: F = 4.03 *; between second and third measurements: F = 7.78 *; PD/analytic groups: F = 3.00 *; analytic/CG, non-significant differences; six-month follow-up: analytic/PD: F = 4.36 *.	Pre/middle/post/ 6-month follow-up
Karataş, 2014 [75]	Community	Bienestar subjetivo; desesperanza	SWS BHS	PD/GC post: Subjective well-being: U = 0.00*; Hopelessness: U = 10.00 *. PD/PG post; Subjective well-being: U = 0.00 *; Hopelessness: U = 2.50 *. Follow-up. Effect was not long-term in subject well-being (*z* = −2.83) *; effect was a long term in hopelessness (*z* = −0.93).	Pre/post/seg. 2.5-month follow-up
Dehnavi et al., 2016 [61]	Clinical	Quality of life	SF-36	PD/CG: Quality of life: F = 93.84 ***.	Pre/post
Kähönen et al. 2016 [71]	Subclinical	Burnout, psychological well-being	BBI SPWB	PD/CG post: autonomy: F = 3.17 **; personal growth: F = 3.03 **. PD/analytic group post: mean change: F = 3.92 ***; Purpose in life: F = −3.36 **. PD/analytic group, follow-up mean change: F = −3.10 **.	Pre/post
Özbas y Tel, 2016 [72]	Subclinical	Psychological empowerment, work empowerment, burnout	PES CWEQ–II MBI	Empowerment, pre/post/follow-up: F = 24.00 ***, structural empowerment, pre/post/follow-up: F = 3.86 *; emotional exhaustion, pre/post/follow-up: F = 38.55 ***, desensitization, pre/post/follow-up: F = 12.80 ***, personal achievement, pre/post/follow-up: F = 10.34 ***; empowerment, PD/CG: F = 38.00 ***; structural empowerment, PD/CG: F = 3.50; emotional exhaustion, PD/CG: F = 40.33 ***; desensitization, PD/CG: F = 24.08 ***; personal achievement, PD/CG: F = −9.68 ***.	Pre/post/ 3-month follow-up
Dogan, 2018 [74]	Community	Empathic tendency scores	ETS	PD pre/post: ETS: *z* = −3.18 ***; PD/CG post: ETS: U = 28.50 **	Pre/post

Note. M: media. StD: standard deviation; PD: psychodrama; CG: control group; CBT: cognitive behavioral therapy; IG: interaction group; PG: placebo group; Aggression Scale: 34 items; physical aggression, verbal aggression, anger, hostility, indirect aggression, and total aggression (Can, 2002); BBI: Bergen Burnout Inventory (Matthiesen and Dyregrov, 1992), 9 items, exhaustion at work, cynicism toward the meaning of work, sense of inadequacy at work; BDI: Beck Depression Inventory (Beck, Ward, Mendelson, Mock, and Erbaugh, 1961). 21 items, depression; BEES: Emotional Empathy Scale (Mehrabian, 1996), 30 items; individual’s vicarious emotional response to the perceived emotional experiences of others; BHS: Beck Hopelessness Scale (Beck, Weissma, Lesteq, and Tralel, 1974), 20 items, hopelessness; CBQ-20: Conflict Behavior Questionnaire–20 (Robin and Foster, 1989), 20 items, parent-adolescent conflict; CBCL: Child Behavior Checklist (Achenbach and Rescorla, 2001). Oppositional defiant problems scale, 5 items. Total problems scale, 60 items (externalizing, internalizing, social, thought, attention problems subscales, etc.); anxious-depressed problems scale, 13 items; CRBDS: Conflict Resolution Behavior Determination Scale (Koruklu, 1998). 24 items, aggression, problem solving; CRS-R: Conners’ Rating Scales—Revised (Conners, 1997), attention-deficit/hyperactivity disorder (ADHD), conduct disorders, cognitive/inattention problems, family problems, emotional problems, anger control problems, and anxiety problems; CWEQ–II: Conditions for Work Effectiveness Questionnaire–II ((Laschinger et al., 2001), 12 items, perceived access to opportunity, support, information and resources; ECR-R: Experiences in Close Relationships-Revised Form (Fraley, Waller, and Brennan, 2000), 36 items, anxiety and avoidance; ETS: Empathic Tendency Scale (Dökmen, 1988), 20 items, emotional component of empathy and one’s potential for it in everyday life; JI: The Jesness Inventory (Jesness, 1996), 80 items; social maladjustment, value orientation, immaturity, autism, alienation, manifest aggression, withdrawal, social anxiety, repression, denial, and asocial index; MBI: Maslach’s Burnout Inventory (Maslach and Jackson,1981), 22 items, emotional burnout, desensitization, personal achievement; MFFT: Matching Familiar Figures Tests (Kagan, 1965), 12 items; reflective-impulsive dimension; Scores are based on the mean response latency (MFFT Latency) and on the mean number of errors produced (MFFT-Error); OLQ-13: Orientation to Life Questionnaire (Antonovsky, 1987)-13 items, assesses three dimensions: comprehensibility, manageability, and meaningfulness; PDQL: Questionnaire Parkinson’s Disease and Quality of Life (Boer AGE, Wijker, Speelmen, and Haes, 1996), 37 items, on aspects: parkinsonism symptoms, systemics symptoms, emotional functioning, and social functioning; PES: Psychological Empowerment Scale (Spreitzer, 1995), 12 items, meaning, competence, autonomy, and impact; SCL-90-R: Symptom Check List-90-Revised (Derogatis, 1994), 90 items, assesses the different dimensions of the symptoms relating to the different diagnostic categories: somatization (SOM), obsession-compulsion (O-C), interpersonal sensitivity (IntSens), depression (DEP), anxiety (ANX), hostility (HOS), phobic anxiety (PHOB), paranoid ideation (PAR), psychoticism (PSY), GSI Global Severity Index); SEI: Self-Esteem Inventory (Coopersmith, 1989), self-attitudes in four areas (social self-Peers, home-parents, school-academic, and general-self); SF-36: 36-Item Short Form (Ware, 1992), 36 items, physical function, physical role functioning, body pain, social role functioning, emotional role functioning, general health perceptions, vitality, and mental health; SPWB: Scales of Psychological Well-Being (Ryff, Lee, Essex, and Schumutte, 1994). 84 items, self-acceptance, positive relationships, autonomy, domain of the environment, purpose in life, and personal growth; STAI: State-Trait Anxiety Inventory (Spielberger; Gonuch, Leushene, Egg, and Jacobs, 1983), 40 items, state anxiety and trait anxiety; SWS: Subjective Well-Being Scale (Tuzgöl-Dost, 2005), 46 items, well-being subjective; YSR: Youth Self-Report Form (Achenbach, 1991): 112 items; depression, verbal aggression, delinquent behavior, thought problems, somatic complaints, social (withdrawn) problems, attention-seeking behavior, and phobic-anxious behavior; * *p* < 0.05, ** *p* < 0.01, *** *p* < 0.001.

### 3.3. Methodological Characteristics of the Research Carried Out

This section summarizes the results obtained in relation to the assessment instruments, variables and their measurement, the substantive variables of the samples and the characteristics of the interventions.

Assessment instruments, variables, and measures. Table 3 outlines the assessment instruments used and the variables measured in each study. It also specifies the follow-up measures established in each case. All trials included at least two measures, pre and post; moreover, the works by Karataş and Gökçakan [62,67,68], Özbaş and Tel [72], Smokowski and Bacallao [69], and Kähönen et al. [71,73] also included long-term follow-up measures.

Substantive variables of the samples. Table 4 shows the results for the sociodemographic variables age and sex. In over half of the studies selected, participants were under 20 years of age [62,63,65,67,68,69,70], although interventions were also carried out with people aged between 20 and 40 [64,72,74] and even with those aged 50 and over [61,71,73]. In all cases, psychodrama was applied to both men and women. As regards sample size, the mean figure was 40.12 individuals per trial. The study by Gatta et al. [63] was the smallest in terms of sample size, with just 12 participants, and the largest were those conducted by Kähönen et al. [71,73], with over 90 people, and Smokowski and Bacallao [67], with 81 families.

Characteristics of the interventions. Table 4 explains how the sessions were structured (number and duration of the sessions, interval between sessions, and length of the intervention) and the type of treatment applied to each group. The mean number of sessions was 19.69, the mean duration of each session was between 106′ and 118′, and the interval between sessions was seven days. Nevertheless, in the two studies by Kähönen et al. [71,73], the interventions were completely different, since four sessions were scheduled each treatment day, with treatment days being separated by an interval of two weeks, up to a total of 32 weeks, which was the overall duration of the intervention. As regards the type of treatment, psychodrama group therapy was implemented in all cases. Two types of treatment were provided to the active control groups: the group analytic method in the studies conducted by Kähönen et al. [71,73] and cognitive behavioral therapy in the trial led by Karataş and Gökçakan [67]. The group analytic method is based on free-floating discussion, similar to the “free association” of classic psychoanalysis. Passive control groups were subjected to similar conditions without treatment, described as follows by the different authors: waiting list, control groups, placebo, and support groups.

## 4. Discussion

The general aim of this systematic review was to identify evidence of psychotherapeutic interventions based on classic psychodrama applied within the framework of controlled clinical trials and published up until December 2019.

The first specific aim was to determine the scientific production and quality of the controlled trials designed to evaluate the efficacy or effectiveness of psychodrama. To this end, the review combined systematic analysis of a broad range of bibliographic sources with the manual review of references found in the documents returned by the search. The validity of a review depends, to a certain extent, on the documentary sources selected, and according to cost-effectiveness analyses carried out in previous studies, e.g., [76], these sources must be selected in accordance with document coverage, topic, geographical area, and language. Thus, for example, in order to avoid publication bias, one of the most important products of the ProQuest database was included, namely ProQuest Dissertations and Theses, which constitutes one of the largest collections of doctoral thesis in the world. Similarly, the contribution made by an open access resource, PubPsych, is also worth mentioning. PubPsych offers access to databases from all over Europe, thereby counteracting one of the most common biases in systematic reviews, the English-speaking bias, which is the result of the greater number of documents written in English [76].

Having chosen the documentary sources, the next step was to decide on the search terms. Here, it is worth highlighting the importance of including a set of terms broad enough to facilitate suitable information collection, despite the possibility of registering false positives. Thus, after removing duplicates, 2960 references were returned which complied with the pre-established search equation. Documents deemed ineligible included studies with no control group, trials which used only one specific psychodrama technique, exclusively qualitative studies and controlled trials in which psychodrama was implemented in conjunction with another type of treatment. Furthermore, we also detected a large number of non-relevant studies (31.62% of the initial references returned by the search) related to other topics such as sociodrama, drama therapy, play drama and different artistic or creative methods, etc. Although they use similar techniques, these methods do not correspond fully to what scientific literature understands to be classic psychodrama, which was the object of our study. The high number of non-relevant references returned may be due to an incorrect categorization of metadata by documentalists or to an erroneous choice of key words by authors, who failed to take into account the scientific evolution of the term “psychodrama”.

Focusing on the results obtained in relation to scientific production, only 143 references (4.69%) were empirical studies carried out with a control group. Thus, in the specific field on which this review focused, we found that only a few clinical trials have attempted to provide evidence of how psychodrama may affect the treatment of mental problems. Up until the 21st century, publications on psychodrama tended to be descriptive or quantitative studies lacking in methodological rigor [33,34]. Nevertheless, over the last two decades, the number of trials carried out with control groups has increased, as shown by the fact that 14 of the 15 studies which fulfilled the inclusion criteria fell into this category. The 15 works selected were written by 11 different research teams, and 66.66% were co-authored, a result which reflects a consolidated tendency toward collaboration. Although no publications stemming from international collaboration were identified, the majority involved collaboration between two different centers, most frequently between a psychotherapeutic intervention clinic and a Higher Education institution.

Although the number of works included in the final review was low, seven were found to have a quality analysis rating of “strong”, and the remaining eight were classified as “moderate” [61,62,63,64] in accordance with the EPHPP criteria [59]. In general, the measures for each of the dimensions evaluated (selection bias, study design, confounders, blinding, data collection method, withdrawals and drop-outs, intervention integrity, and analyses) reveal that all 15 studies had a high level of methodological rigor. In relation to blinding, the controlled trials selected did not usually specify whether the evaluator was aware of the experimental condition; however, it should be remembered that the double-blind requirement in Psychology is difficult to guarantee when the psychotherapist (who is aware of the nature of the treatment being provided) and the evaluator are the same person. This lack of information was also reflected in other dimensions, such as selection bias or the identification of key differences between groups prior to the intervention. In contrast, in all cases, information was provided on the percentage of participants completing the study. Intervention results cannot be generalized in the event of participant losses of over 20% [77] (in such cases, patients are considered to be non-adherent). In 93.33% of the publications reviewed, however, a lost follow-up rate of less than 20% was reported, meaning that the participants can be considered adherent. The methodological rigor of the controlled trials was consistent with the quality of the journals in which they were published since 73.33% were indexed in either Scimago Journal and Country Rank (SJR) or Journal Citation Reports (JCR).

The second aim of this systematic review was to determine what types of problems were treated using group psychodrama therapy. Firstly, we analyzed the studies involving clinical patients with clinical symptoms. Thus, in adolescents diagnosed with oppositional defiant disorder (impulsivity and oppositional conduct), a significant change was observed in the behavioral variables of the group treated with psychodrama, although no improvement was noted in variables measuring empathy and self-esteem, which would require more time than the 12 weeks for which the treatment lasted [65]. We also observed an improvement in operationalized impulsivity due to a greater response latency and fewer errors produced. This improved response latency was due not to greater fear of failure or the inability to come up with alternative solutions but rather to greater reflection. Adolescents learned to consider the consequences of their actions and to respond more responsibly. The reflective style seemed to reduce the aggressiveness and oppositional conduct, which formed part of their impulsivity and lack of planning [65]. Moreover, the authors claimed that psychodrama helped patients get to know themselves better; following treatment, subjects were better able to connect to their emotions and feelings in a safe therapeutic setting; moreover, they tested different roles and learned a new repertoire of responses and behaviors [78,79]. The process of gaining greater emotional control was also a key factor in the results reported by Sproesser et al. [66] when treating patients with Parkinson’s disease. In this case, psychodrama helped participants reorganize their daily routine, establish social ties and reduce anxious-depressive symptoms, all of which had a positive effect on their psychological well-being (as a measure of quality of life). This perspective was also shared by Dehnavi et al. [66], who highlighted the fact that psychodrama brought about changes in cognitive insight, consciousness level [...] depth and scope of individual experiences, understanding [of] self-strengths and weaknesses which are key to improving quality of life. The therapeutic techniques selected and the work of the therapist are essential in this sense since they aid patients in coping with their psychological distress, creating a space for self-reflection, which helps them feel more understood [80]. Patients not only feel that others understand them better but also discover that they are not alone and that their peers are struggling to cope with the same problems, thereby creating what Foulkes calls a “mirror reaction” [81].

Secondly, we analyzed those trials in which psychodrama was applied to people suffering from a certain degree of psychological distress, but without reaching the level required for their problem to be considered psychopathology. In these cases, psychodrama was used to help them control their aggressive impulses and defiant conduct (e.g., impulsivity, defiant behavior, parent-adolescent conflicts, etc.) or to reduce the distress caused by the presence of chronic stressors. In studies focusing on aggressiveness, many therapists have used intervention programs in both clinical practice [82] and controlled research trials [4,12,32,83]. The results of the clinical trial conducted by Karataş and Gökçakan [68] revealed a decrease in participating adolescents’ overall aggressiveness scores. Specifically, measures for anger, hostility, and indirect aggression were observed to decrease, although physical and verbal aggression scores remained the same. According to the authors, one possible reason for this absence of change in physical and verbal aggression may have been that some of the young people in the group continued to receive disciplinary punishment at school, something which may have contributed to the maintenance of their aggressive reactions. Moreover, verbal aggression is a type of behavioral manifestation that is a common phenomenon in participants’ daily lives. Symptoms associated with chronic stress constitute another focus of interest in psychodrama interventions. In the study conducted by Carbonell and Parteleno-Barehmi [70] with girls coping with traumatic-stress exposure, the two areas in which significant changes were observed as a result of the treatment were anxiety/depression and social withdrawal (isolation). These are particularly sensitive areas in the success of therapies for trauma and chronic stress. In this sense, psychodrama may potentially play a key role in the treatment of post-traumatic stress. The study also highlights the importance of group therapy for helping participants to develop better coping strategies and to gain a greater sense of competence [70]. Psychodrama has also been used to treat one of the most common types of chronic stress found in the workplace: burnout. Burnout is usually defined as a syndrome characterized by three dimensions [84]: emotional exhaustion, depersonalization, and lack of personal accomplishment. Over recent years, this question has awakened great interest among psychodrama specialists studying the care or helping professions [71,72,73,85,86]. Kähönen et al. [71,73] assessed burnout through two modulating variables: sense of coherence and psychological well-being, both of which mitigate burnout and are particularly effective for preventing emotional exhaustion, always under the hypothesis of an inverse relationship existing between the two constructs. The authors highlight the importance of providing this kind of group therapy since it is a cost-effective intervention in the context of medical care; thus, treatment with psychodrama may have a major impact on workers’ mental health.

Thirdly, we analyzed cases in which psychodrama had been used with community samples to explore how certain variables may influence mental health or professional competencies. The study by Dogan [64] analyzed the effect of group psychodrama on romantic attachment styles among adults. The results failed to reveal any changes in the variables studied (avoidance and anxiety), probably because a longer treatment time is required to improve maladaptive attachment styles. Nevertheless, following the intervention, participants reported improvements in empathy and their ability to react reliably and also said they were more aware of their strengths and weaknesses. Another particularly important variable in the healthcare field is hopelessness, a construct that promotes a distorted view of reality and is believed to predict suicide [87]. In her study, Karataş [75] focused directly on subjective well-being and hopelessness, finding effective evidence of an improvement in both variables directly after the intervention, although, in the follow-up test, only the effect of the treatment on hopelessness was found to have persisted. Although further studies are required to corroborate these results, this is a very promising avenue of research due to its implications for mental health, since psychodrama has a beneficial effect on certain positive variables such as social adjustment, quality of life, self-awareness, subjective well-being, and psychological well-being. Dehnavie et al. [61] reported that the psychodrama intervention increased personal exchanges and served as indirect training for social skills; it also resulted in a greater depth of individual experiences and a better understanding of self-strengths and weaknesses, thereby fostering emotional and cognitive integrity [9].

The authors of primary studies report benefits for both patients with clinical and subclinical symptoms and participants from community samples. These findings are consistent with the results of previous meta-analyses, which found significant improvements in all types of participants, although those with subclinical symptoms were found to benefit more from psychodrama, obtaining a slightly larger effect size [25,31]. The capacity of psychodrama to adapt and be applied to any environment or population type has been widely documented in the literature [32,88].

Finally, the third aim was to explore the methodological characteristics of the research studies carried out. The studies included in the systematic review used a wide variety of measures. Psychodrama was found to influence dependent variables such as symptoms, subjective well-being, quality of life, and manifest behavior, among others, measured using self-reports. This type of assessment instrument may generate bias in the measurements due to a lack of motivation by participants, possible simulation, social desirability, lack of introspection skills and memory distortion, etc. see [89], and these biases may, in turn, affect the reliability of the conclusions drawn. Nevertheless, six of the publications followed mixed designs, meaning that the information obtained through qualitative interviews was used to complement and explain the quantitative results. In relation to the follow-up tests administered after the therapy, in the case of psychosocial interventions, and particularly psychodrama, it is best to take measures after an interval of (at least) two weeks to provide participants time to process the experience [72]. Nevertheless, in eight of the 15 studies included in the review, assessments were only carried out immediately after the end of the intervention. In this sense, follow-up evaluations should always be carried out in order to corroborate the long-term efficacy of psychodrama. In relation to the type of participants undergoing treatment, most were young people under the age of 20. As regards sample size, the groups were generally small [63,66] which, while rendering them more manageable in terms of the psychotherapeutic intervention itself, nevertheless prevents the results from being generalized.

One of the methodological inclusion criteria for this review was that studies distinguish between the three classic phases of psychodrama conceptualized by Moreno: warming up, action and sharing. The first phase is designed to generate trust and get the group into the right mood for the different temporal, spatial, and relational movements [63] that occur during the action. During the second phase, the problem itself is addressed and alternatives sought. It is during this phase that the different psychodrama techniques are applied, and participants experience catharsis. Finally, during the sharing phase, participants identify with what has occurred and share feelings and experiences. Thus, both the group and the protagonist benefit from the exchange. Nevertheless, in the studies analyzed, the content of each phase was influenced, to a large extent, by the different people and elements involved in the psychodrama sessions, including the group itself, the problem being treated and the decisions made by the session leader. In this sense, it is important to highlight the fact that, in order for a group intervention to be effective and safe, therapists must be properly qualified and trained.

Upon analyzing the structure of the sessions and the duration of the interventions, it becomes clear that it is difficult to establish a rigorous, stable, structured pattern for applying psychodrama therapy. Interventions generally encompassed 12 sessions (although they varied between eight and 64) and lasted for around four months. Most individual sessions lasted 1–2 h. Despite the positive changes observed following the treatment, in most cases, the authors highlight the need for longer interventions in order to render the effects more stable [61,64,75]. This idea is consistent with the idiosyncrasy of psychodrama itself since it takes time to generate the required catharsis and then assimilate and integrate it. In this sense, although Kipper and Ritchie [25] argue that the number of sessions does not affect the efficacy of the techniques, it remains to be seen whether it affects the efficacy of the intervention as a whole.

This systematic review has some limitations, the main one being the low number of randomized controlled trials that have been published in this field. It is important to encourage and conduct research designed to evaluate the effectiveness of the intervention, comparing the results with those obtained in other groups not receiving treatment. This would help validate the method and enable the benefits of psychodrama to be felt in a multitude of different scenarios. Nevertheless, not only is it important to design methodologically robust controlled trials, but it is also necessary for the resulting reports to include sufficient information to enable the studies to be replicated. The EPHPP (Effective Public Health Practice Project) [59,60] has proven to be a vital tool in this sense, enabling the quality of primary studies to be assessed and the gaps and weak points of each publication to be identified. For example, 40% of the documents analyzed here failed to state whether or not there were important differences between the group at the start of the intervention; 66.66% failed to specify whether or not the sample was representative; and 47.7% of the trials failed to determine the competence level of the psychodrama therapist. Therapist competence is key to determining the reliability of the implementation, and PD practitioners should have the credentials required to lead the sessions effectively, although it is also true that, to date, all training programs have been run by non-academic professional private associations and institutions [24]. Returning to the idea of the importance of replicability, RCT reports should follow clear guidelines for improving research transparency. The CONSORT (Consolidated Standards of Reporting Trials) group has drafted an extension to its original declaration (the CONSORT extension for NPT trials) that focuses on specific methodological issues linked to RCTS involving non-pharmacological treatments, such as psychotherapeutic interventions [90]. One of the recommendations made is that reports include a flow chart (specifying the number of people participating in each trail phase: enrollment, allocation, follow-up, and analysis) and checklists as tools for ensuring compliance with the required standards of quality and scientific rigor. Following these guidelines would improve the comprehensibility (and therefore replicability) of the RCTs published.

Further research is also required in the field of work-related health and in biohealth contexts, with an effort being made to structure the intervention as much as possible (in a systematic and replicable manner) within, of course, the boundaries of the idiosyncrasy of psychodrama itself, which is based on improvisation and adaptation to each individual situation and patient.

Future research may wish to increase the number of randomized controlled trials in order to ensure that psychodrama can be applied within public health service, thereby benefiting a greater number of people. To this end, an effort should be made to ensure that the treatment is applied in as systematic a manner as possible. We also recommend that future studies work with samples that are representative of the population and that they increase the number of psychodrama sessions, carry out follow-up assessments in all cases and use, alongside self-reports, other evaluation instruments that complement the results obtained and help explain and understand them. Alongside result-centered research (which seeks to ascertain whether or not therapy leads to change), it is also important to conduct process-centered research, focusing on elements such as the identification, determination, and relationship between common and specific factors (e.g., participant expectations, therapeutic alliance, the group process itself, significant events research, insight, self-awareness, links between clients’ in-session dramatic engagement, and concretization, etc.). According to recent findings [39,91,92,93], these factors all contribute to the mechanisms of change at work during therapy sessions.

To conclude, we can state that psychodrama offers a holistic view of human psychology, works with corporal memory, and the sensations experienced by the physical body offer a possible restructuring of the conflicting parts of each individual and, in short, may help patients in a wide variety of different situations. Psychodrama has been found to be beneficial for most of the variables analyzed and is an effective means of reducing certain symptoms and fostering certain strengths and positive attitudes. We hope that this review will help boost research in this field, increasing its scientific validity and rendering the use of this therapy more widespread, thereby improving people’s quality of life.

## Figures and Tables

**Table 1:** Bibliometric data pertaining to the retrieved references.

Year	Authors	Affiliation	Country	Doc. Type	Journal	Quality Indicators
1999	Carbonell, Dina M.	Bridgewater State University	USA	Article	International Journal of Group Psychotherapy	SJR (99) = Q2: 0.429.
	Parteleno-Barehmi, Ceil	Wheelock College, Boston				
2003	Singal, Sally	McGill University	Canada	Thesis		
2009a	Karataş, Zeynep	Mehmet Akif Ersoy University	Turkey	Article	Educational Sciences: Theory & Practice	SJR (09) = Q4: 0.111.
	Gökçakan, Zafer	Mersin University				
2009b	Karataş, Zeynep	Mehmet Akif Ersoy University	Turkey	Article	Turkish Journal of Psychiatry	
	Gökçakan, Zafer	Mersin University				
2009	Smokowski, Paul R.	U. of North Carolina at Chapel Hill	USA	Article	Small Group Research	SJR (09) = Q2: 0.763. JCR (09) = 0.683
	Bacallao, Martica	U. of North Carolina at Greensboro				
2010	Dogan, Turkan	University of Baskent	Turkey	Article	Arts in Psychotherapy	SJR (10) = Q3: 0.256. JCR (10) = 0.609
2010	Gatta, Michela	University of Padua	Italy	Article	Arts in Psychotherapy	SJR (10) = Q3: 0.256. JCR (10) = 0.609
	Lara, Dal Zotto	University of Padua				
	Lara, Del Col	ULSS 16 Padua				
	Andrea, Spoto	University of Padua				
	Paolo, Testa Costantino	ULSS 16 Padua				
	Giovanni, Ceranto	ULSS 16 Padua				
	Rosaria, Sorgato	ULSS 16 Padua				
	Carolina, Bonafede	ULSS 16 Padua				
	Pier Antoni, Battistella	ULSS 16 Padua				
2010	Sproesser, Erika	University of Campinas	Brazil	Article	Parkinsonism and Related Disorders	SJR (09) = Q1: 1.05. JCR (09) = 2.406
	Viana, Maura A.					
	Quagliato, Elizabet M.A.B.					
	de Souza, Elisabete A. P.					
2011	Karataş, Zeynep	Mehmet Akif Ersoy University	Turkey	Article	Educational Sciences: Theory & Practice	SJR (11) = Q3: 0.200.
2012	Kähönen, Kari	University of Jyväskyla	Finland	Article	Personality and Social Psychology	SJR (12) = Q1: 5.689. JCR (12) = 4.877
	Naatanen, Petri	University of Jyväskyla				
	Tolvanen, Asko	University Central Hospital				
	Salmela-Aro, Katariina	University of Helsinki				
2014	Karataş, Zeynep	Mehmet Akif Ersoy University	Turkey	Article	Eğitim ve Bilim	SJR (14) = Q3: 0.276.
2016	Dehnavi, Saeed	Islamic Azad University	Iran	Article	International Journal of Medical Research & Health Sciences	
	Bajelan, Mahin					
	Pardeh, Setareh Javaher					
	Khodaviren, Hamideh					
	Dehnavi, Zahra					
2016	Kähönen, Kari	University of Jyväskyla	Finland	Article	Psykologia	
	Muotka, Joona	University of Jyväskyla				
	Näätänen, Petri	University of Jyväskyla				
	Salmela-Aro, Katariina	University of Helsinki				
2016	Özbaş, Azize Atli	Hacettepe University	Turkey	Article	Palliative and Supportive Care	SJR (16) = Q2: 0.500. JCR (16) = 1.199
	Tel, Havva	Cumhuriyet University				
	Azoulay, Bracha	University of Haifa				
	Snir, Sharon	Tel Hai College				
	Regev, Dafna	University of Haifa				
2018	Dogan, Turkan	Hacettepe University	Turkey	Article	PsyCh Journal	SJR (18) = Q3: 0.401. JCR (18) = 0.717

Note: SJR (Scimago Journal and Country Rank), JCR (Journal Citation Reports).

**Table 2:** Quality assessment tool for quantitative studies (Wess et al., 2012).

Authors	Year	G1	SD	G2	Ca	G3	G4	G5	Wa	Wb	G6	Ia	Ib	Aa	Ab	Ac	GG
Carbonelly and Parteleno-Barehmi	1999	**++**	RCT	**+++**	No	**+++**	**++**	**+++**	Yes	82%	+++	93%	Yes	Middle School	Yes	Yes	**+++**
Singal	2003	**+++**	RCT	**+++**	No	**+++**	**++**	**+++**	Yes	100%	+++	100%	Yes	High School	Yes	Yes	**+++**
Karataş and Gökçakan	2009a	**++**	RCT	**+++**	No	**+++**	**++**	**+++**	Yes	97.2%	+++	96.0%	CT	High School	Yes	Yes	**+++**
Karataş and Gökçakan	2009b	**+++**	RCT	**+++**	No	**+++**	**+**	**+++**	Yes	95.8%	+++	91.6%	Yes	High School	Yes	Yes	**++**
Smokowski and Bacallao	2009	**+++**	RCT	**+++**	No	**+++**	**++**	**+++**	Yes	CT	++	CT	Yes	Latino communities	Yes	Yes	**+++**
Dogan,	2010	**+**	RCT	**+++**	CT	**+++**	**++**	**+++**	Yes	65%	++	69%	CT	University	Yes	Yes	**++**
Gatta et al.	2010	**+**	q-RCT	**+++**	No	**+++**	**+**	**+++**	Yes	100%	+++	100%	Yes	Public Health Services	Yes	Yes	**++**
Sproesser et al.	2010	**++**	RCT	**+++**	CT	**+++**	**++**	**+++**	Yes	100%	+++	100%	CT	University Hospital	Yes	No	**+++**
Karataş	2011	**+**	RCT	**+++**	No	**+++**	**++**	**+++**	Yes	100%	+++	100%	CT	High School	Yes	Yes	**++**
Kähönen et al.	2012	**++**	RCT	**+++**	CT	**+**	**++**	**+++**	Yes	82%	+++	79%	CT	Healthcare Service	Yes	Yes	**++**
Karataş	2014	**++**	RCT	**+++**	No	**+++**	**++**	**+++**	Yes	100%	+++	100%	Yes	University	Yes	Yes	**+++**
Dehnavi et al.	2016	**++**	RCT	**+++**	CT	**+++**	**++**	**+++**	Yes	CT	+	CT	CT	Addiction Treatment Clinic	Yes	No	**++**
Kähönen et. al.	2016	**++**	RCT	**+++**	CT	**+**	**++**	**+++**	Yes	100%	+++	100%	CT	Occupational Health Care	Yes	Yes	**++**
Özbas and Tel	2016	**++**	RCT	**+++**	No	**+++**	**+**	**+++**	Yes	90%	+++	78.9%	Yes	University Hospital	Yes	Yes	**++**
Dogan	2018	**+++**	RCT	**+++**	CT	**+**	**++**	**+++**	Yes	100%	+++	100%	Yes	University	Yes	Yes	**+++**

Note: +++: strong, ++: moderate, +: weak. RCT: randomized controlled trial; q-RCT: quasi-randomized controlled trial. CT: cannot tell. Selection bias: G1 (global rating selection bias). Design: SD (study design) and G2 (global rating study design). Confounders: Ca (Were there important differences between groups prior to the intervention?) and G3 (global rating confounders). Blinding: G4 (global rating blinding). Data collection methods: G5 (global rating data collection methods). Withdrawals and drop-outs: Wa (Were withdrawals and drop-outs reported in terms of numbers and/or reasons per group?), Wb (Indicate the percentage of participants completing the study (if the percentage differs by groups, record the lowest)), and G6 (global rating withdrawals and drop-outs). Intervention integrity: Ia (What percentage of participants received the allocated intervention or exposure of interest), Ib (Was the consistency of the intervention measured? Consistency here is understood here as therapist adherence (not compromised by being a group intervention) and therapist competence, assessed in terms of informed expertise, with “yes” indicating the maximum level), Analyses: Aa (Indicate the unit of allocation: community, organization/institution, practice/office, individual), Ab (Are the statistical methods appropriate for the study design?), and Ac (Is the analysis performed by intervention allocation status (i.e., intention to treat) rather than the actual intervention received?). GG: global rating for this paper.

**Table 4:** Substantive variables of the sample (age and sex), structure of the sessions (number and duration, interval between sessions, and length of the intervention) and type of treatment.

Study	Sample			SD	Session Structure				Treatment			
	Sex		Age									
	w	m			NSD	TNS	IBS	LOI	EG	(n)	CG	(n)
Carbonell y Parteleno-Barehmi, 1999 [70]	26	0	11–13	N.A.	N.A.	N.A.	N.A.	20	PD	(12)	WL	(14)
Singal, 2003 [65]	18	6	12–17	60–120′	1	12	7	12	PD	(13)	WL	(11)
Karataş y Gökçakan, 2009a [67]	13	23	13–14	90–120′	1	14	7	14	PD CBT	(12) (12)	CG	(12)
Karataş y Gökçakan, 2009b [68]	12	11	13–14	90–120′	1	14	7	14	PD	(11)	CG	(12)
Smokowski y Bacallao, 2009 [69]	54	27	14	180′	1	8	7	8	PD	(56)	SG	(25)
Dogan, 2010 [64]	15	5	23–29	120′	1	12	7	12	PD	(11)	CG	(09)
Gatta et al., 2010 [63]	4	8	15–18	75′	1	12	7	12	PPD	(06)	CG	(06)
Sproesser et al., 2010 [66]	7	9	49–53	90′	1	12	15	24	PD	(08)	WL	(08)
Karataş, 2011 [62]	18	18	14–17	90–120′	1	10	7	10	PD	(12)	CG IG	(12) (12)
Kähönen et al., 2012 [73]	70	24	31–59	90′	4	64	15	32	PD AG	(30) (32)	CG	(32)
Karataş, 2014 [75]	45		N.A.	90–120′	1	12	7	12	PD	(15)	CG PG	(15) (15)
Dehnavi et al., 2016 [61]	0	30	20–52	120′	1	12	N.A.	6	PD	(15)	CG	(15)
Kähönen et. al., 2016 [71]	70	24	33–59	90′	4	64	15	32	PD AG	(30) (32)	CG	(32)
Özbas y Tel, 2016 [72]	82	0	18–37	120′	1	10	7	10	PD	(38)	CG	(44)
Dogan, 2018 [74]	22	1	23–39	180′	1	12	7	12	PD	(14)	CG	(09)

Note: SD: session duration (min.); NSD: number of sessions per day; TNS: total number of sessions; IBS: interval between sessions, in days; LOI: length of the intervention, in weeks; EG: experimental group; CG: control group; N.A.: not available; PD: psychodrama; WL: waiting list; CBT: cognitive behavioral therapy; SG: support group; AG: analytic group; PG: placebo group; IG: interaction group.

## Data Availability

Not applicable.
